# The Open Air Treatment of Phthisis

**Published:** 1898-10-15

**Authors:** Richard Greene


### The Institutional Workshop.

### THE OPEN AIR TREATMENT OF PHTHISIS.

### A VISIT TO FALKENSTEIN.

By Richard Greene, F.R.C.P.Ed.

Forty years since or more Hughes Bennett pointed out the benefits which consumptive patients sometimes derived from sitting at an open window or from walking round Arthur's Seat in the crisp air of an Edinburgh winter; and later, MacCormack had enunciated similar views in his little book entitled "Consumption and Re-breathed Air." But their words were sown on rocky ground; anyway, not much fruit was produced, and the open air treatment, as now understood, was like much else nowadays—"made in Germany." It was, I believe, Dettweiler, of Falkenstein, who elaborated this system and carried it out to its logical conclusion.

Falkenstein is quite a small village situated in the Taunus Range, and has an altitude of 400 meters above the sea level. It is approached from Frankfort-on-the-Main by train to Cronberg, which takes about forty minutes, and after a drive of like duration the hospital is reached. There is a gradual ascent all the way from Cronberg, and the road winds through very pretty woodland scenery. Close in sight is one of the country seats of our own Princess Royal, and many bright-looking villas are passed on either side. Königstein is about half an hour's drive, and Homburg about one hour's from Falkenstein.

The entrance to the building stands close to the village street, and is to the north, an arrangement which gives the main façade of the hospital a southern aspect, and places the whole of the beautiful grounds, about 80 acres, away from the public roads, and ensures privacy. The building consists of a centre, which is the older part built in 1874, and of two wings, which are of recent date. The administrative and commissariat departments are well arranged; and the whole pile of buildings, land, planting, roads, and engineering are said to have cost £100,000. It was erected by some philanthropists of Frankfort, who, however, wisely treated it as a money investment. After a profit of 5 per cent. per annum has been distributed to the shareholders, the rest of the profit goes to improve the hospital or to help patients in a neighbouring institution.

On the day of my visit there were 120 patients in residence; three of these were English, six French, and the rest Germans. The entire staff reaches the very respectable total of 92. The general plan of the building somewhat resembles the letter [illegible] turned so that the usual right hand side of the letter faces almost due south, and from the east and west sides corridors run, at the ends of which the wings are placed. The south side of the central block is surrounded by a broad verandah, around which rows of sofas and lounges of the deck-chair pattern are placed, and on these the patients spend almost the entire day. The verandah can, to some extent, be closed by curtains; but not one of these was drawn on the day of my visit, although the sky was clouded and the air sharp. Kiosks and summer-houses are thickly scattered about the grounds; but most of them are at no great distance from the hospital, and none are provided with doors or other means of being closed. On the verandahs, in the corridors, in the walks, and throughout the grounds are placed spittoons, and smaller ones are carried by the patients, who are forbidden to expectorate on the floors, walks, or roads. Any breach of this rule involves the discharge of the patient.

The patients rise at eight in the morning and go to bed at ten at night, and, excepting the time spent at meals, the whole of the 14 hours must be passed out of doors regardless of the state of the weather. On going to bed the windows are sometimes closed until the patient is in bed but they are invariably opened afterwards. Under the windows are coils of steam or hot-water pipes which could be used to warm the incoming air, if the external temperature fell very low. The bed-room floors are covered with linoleum, and articles of furniture likely to harbour dust are few. The diet is, of course, liberal, and the hours for meals are: Breakfast, eight a.m.; dinner, one p.m.; and supper, seven or half-past seven p.m. The patients are encouraged to drink milk, and to eat much fat and butter. Except in rare cases animal food is given *ad lib.* Bread, green vegetables, and stewed fruit are largely consumed, and potatos sparingly. Light German wines are almost invariably used with the two chief meals. The vast majority of the patients take their meals in the large and handsome hall; but there are smaller dining-rooms, which can be used for such patients as cough much, or otherwise may need separation.

There is no special medical treatment in use, and it may be summed up that the Falkenstein system consists of open air, rest, good diet, and careful supervision. As regards the latter it is admirably carried out, and the slightest increase of cough or the appearance of any untoward symptom leads to immediate and thorough investigation, and to the application of appropriate treatment.

As to the "rest" part of the treatment it seemed to me to be almost absolute, for the number of patients I saw walking in the grounds was extremely small. Here, I was informed, there is considerable difference from the treatment carried out at the sister hospital at Ruppershain, where patients ramble about the woods; but I was also informed that at Ruppershain only mild cases are received. The latter hospital is about four miles from Falkenstein, and Dr. Nahm is the director.

The medical officer at Falkenstein, from whom I obtained most of my information, did not set much store on mere climatic conditions, nor even on altitude. Granted that the other conditions are present, these need not have undue weight attached to them. So he seemed to hold, and certainly the mere climate of Falkenstein cannot have any great influence, for the weather is cold enough at times and rain often falls. On the other hand Professor Leibermeister, of Tübingen, believes much in a high altitude, and he thinks the diet of consumptive patients should be more "starchy" than "animal." But every system must be measured by its results; and it is claimed at Falkenstein that the recoveries range from 25 to 30 per cent., and many others are improved. It is admitted that most of these recoveries take place in patients who have not gone beyond the first stage of the disease. But a fair proportion (I did not obtain exact figures) are of cases in the second stage, and cures are by no means unknown even in the third stage. The shortest period which a patient has to remain at Falkenstein is 100 days; but it has often to be prolonged to five or six months. The season of the year seems to make almost no difference, and cures are at least as numerous in the winter as in the summer.

As regards the risk of healthy people being infected by the diseased, I learnt that such a calamity was practically unknown, and that the staff incurred no danger whatever. As the medical officers take their meals with the patients, and associate much with them, it may be presumed that they do not attach any importance to the chance of infection. Of course, extreme care is taken. For instance, no food of any kind which has once been placed on the table is ever used again, and the cleansing of all kinds of utensils and washing of clothes are carried out in the most thorough manner.

The cost of living at Falkenstein varies a little. The board is the same for all, namely, 8s. a day, and the rooms range from about 2s. a day up to about 12s. Medical attendance is included. Wines are supplied at prices lower than in most hotels. There are a few extra payments, but they are very small, and practically the only serious one would be the engagement of a special nurse, and this is so rarely required that it may be left out of sight.

As patients' friends are not allowed to sleep in the hospital, it may be as well to say that lodgings may be obtained in the village, and that there is an hotel (Frankfurter-Hof) better than might be expected from the size of the place.

Dr. Dettweiler has three qualified assistants, and an inspection of the physiological and chemical laboratories showed me that much work was done in these departments, and that in every way the patients were most carefully and skilfully attended to; indeed, it may be fairly surmised that the good results are in great part due to this and to the fact that the patients are under strict discipline and are beyond the reach of interfering friends.

The open-air treatment of phthisis has borne the test of something like a quarter of a century in Germany, but it is only now being seriously talked of in England. So far as a long and fairly exhaustive visit to Falkenstein can be relied on, I can see no reason why the system, in its entirety, should not be carried out as well in England as abroad. There must be many favoured spots where hospitals could be erected. To attempt the system in the patient's own home would probably end in dismal failure.

